# Automatic emotion and attention analysis of young children at home: a ResearchKit autism feasibility study

**DOI:** 10.1038/s41746-018-0024-6

**Published:** 2018-06-01

**Authors:** Helen L. Egger, Geraldine Dawson, Jordan Hashemi, Kimberly L. H. Carpenter, Steven Espinosa, Kathleen Campbell, Samuel Brotkin, Jana Schaich-Borg, Qiang Qiu, Mariano Tepper, Jeffrey P. Baker, Richard A. Bloomfield, Guillermo Sapiro

**Affiliations:** 1 Department of Psychiatry and Behavioral Sciences, Duke Health, Durham, USA; 2 Department of Psychiatry and Behavioral Sciences, Duke Center for Autism and Brain Development, Duke Institute for Brain Sciences, Durham, USA; 3 Department of Electrical and Computer Engineering, Duke University, Durham, USA; 4 Department of Pediatrics, Duke Health, Durham, USA; 5 Department of Biomedical Engineering, Department of Computer Sciences, Department of Mathematics, Duke University, Durham, USA; 6 Present Address: Department of Child and Adolescent Psychiatry, NYU Langone Health, Adjunct at Duke Health, Durham, USA; 7 Present Address: Apple, Inc., Cupertino, USA

**Keywords:** Paediatric research, Neurological manifestations

## Abstract

Current tools for objectively measuring young children’s observed behaviors are expensive, time-consuming, and require extensive training and professional administration. The lack of scalable, reliable, and validated tools impacts access to evidence-based knowledge and limits our capacity to collect population-level data in non-clinical settings. To address this gap, we developed mobile technology to collect videos of young children while they watched movies designed to elicit autism-related behaviors and then used automatic behavioral coding of these videos to quantify children’s emotions and behaviors. We present results from our iPhone study Autism & Beyond, built on ResearchKit’s open-source platform. The entire study—from an e-Consent process to stimuli presentation and data collection—was conducted within an iPhone-based app available in the Apple Store. Over 1 year, 1756 families with children aged 12–72 months old participated in the study, completing 5618 caregiver-reported surveys and uploading 4441 videos recorded in the child’s natural settings. Usable data were collected on 87.6% of the uploaded videos. Automatic coding identified significant differences in emotion and attention by age, sex, and autism risk status. This study demonstrates the acceptability of an app-based tool to caregivers, their willingness to upload videos of their children, the feasibility of caregiver-collected data in the home, and the application of automatic behavioral encoding to quantify emotions and attention variables that are clinically meaningful and may be refined to screen children for autism and developmental disorders outside of clinical settings. This technology has the potential to transform how we screen and monitor children’s development.

## Mobile technologies: revolutionizing behavioral assessments of young children

A phone-based app that assesses the behavior of young children in their homes can be used to determine their risk of autism. Autism is the most common neurodevelopmental disorder in the US. Although some of the signs of autism can be identified within the first months of life, many children wait years to be diagnosed. A study led by Richard Bloomfield, Geraldine Dawson, Helen Egger, and Guillermo Sapiro, Duke University, examined caregiver-collected smartphone videos that quantify the emotion and attention of children aged between 1 and 6 years watching movies known to elicit autism-related behaviors. Automatic coding identified significant differences in emotion and attention by age, sex, and autism risk status. These findings highlight the feasibility of using this approach to identify autism symptoms and, potentially, those of other behavioral disorders.

## Introduction

Autism spectrum disorder (ASD), affecting 1/68 children in the US,^1^ is the most common childhood neurodevelopmental disorder. While identification and early intervention of ASD are public health priorities, barriers limit families’ access to evidence-based screening for ASD. In the US, the median age that a child is diagnosed with ASD is 4,^1^ despite the fact that we can reliably diagnose children at 24 months.^2^ Many children must wait months or even years for evaluation. The situation is significantly worse in low-resource countries. With increasing evidence that early intervention significantly improves outcomes,^3,4^ there is urgency to bridge the gaps between need and access to care, as well as between science and practice.

Our interdisciplinary team came together to develop accessible, and scalable mobile technology tools to bridge these gaps. Here, we present data from Autism & Beyond, (https://autismandbeyond.researchkit.duke.edu/) an iOS ResearchKit (http://www.apple.com/researchkit/) study using iPhones and new behavioral assessment and analysis framework to test the feasibility of a digital health and data science approach to the assessment of young children’s emotions and behaviors in their homes.

Our work emerges from the recognition that a major barrier to early, evidence-based identification and treatment for ASD is the lack of scalable tools for objectively assessing young children’s observed behaviors and emotions. While caregiver-reported information about a child is important, it is far from sufficient for a comprehensive understanding of the child. Currently, the gold standard observational tool for ASD assessment, the Autism Diagnostic Observation Schedule (ADOS^5^), requires administration by trained professionals in clinical settings, is expensive, and is time-consuming. There is a very limited number of trained professionals, leading to long waiting lists even when physical access is possible; and individual differences in making severity ratings adds further challenges in diagnosis and treatment. The lack of feasible, affordable, and accessible observational tools impacts timely identification, the capacity to track developmental change and the effectiveness of interventions, and clinicians’ and caregivers’ access to evidence-based knowledge of children’s risk for autism and other developmental and mental health challenges.

Tools that rely on professional administration are not scalable in their current form. We need complementary aid tools that capture children’s behaviors in their natural environments, including their homes, schools, and community settings, and can track changes over time (see also “Discussion” section). The capacity to assess children outside of clinical and research settings, to engage parents and other caregivers in the collection of data, and to reach families who cannot access services or participate in research will provide additional/complementary information about children that is more ecologically valid and culturally representative. Better population health data and engagement with caregivers will also help to increase awareness about the public health needs of children and families.

We have previously reported on our initial development of automatic computer vision tools for measuring ASD-related behaviors in infants and toddlers in videotapes of children collected in clinical settings (we address prior work on ASD via manual video coding in the “Discussion” section). We described the development of reliable automatic computer vision tools to measure disengagement of attention and visual tracking from videotaped clinical assessment with the Autism Observational Scale for Infants.^6,7^ In this study, video was obtained with an inexpensive GoPro camera and the expert in the room presented the stimuli. Next,^8–10^ we developed an iPad-based tool and tested its use in pediatric primary settings in conjunction with a digital version of the Modified Checklist for Autism and Toddlers-Revised with Follow-up Questions [MCHAT-R/F^11^]. The iPad’s front facing camera recorded a video of the child watching short video (movie) stimuli designed to elicit ASD-related behaviors. Social-emotional behaviors were automatically coded from the video with our computer vision and analysis tools. We validated the automatic tools,^10^ and showed initial correlations with ASD symptoms, in addition to demonstrating that use of mobile apps can improve questionnaires administration.^8,9,12–14^ The validation of the automatic algorithms, as described in ref. ^10^, was done independently of this study and of diagnosis. We showed that experts and the computer algorithm agree in 74 and 75% of the frames for affect coding, for the control and ASD groups respectively, with most of the differences resulting from those frames at the beginning or end of the coded emotion. The same is found for inter-rated reliability, meaning the automatic algorithm is as close to the experts as the experts among themselves, with virtually no difference in emotion coding but a slight difference in duration (few frames differences). Furthermore, for social referencing (head turning to look at caregiver) the intra-class correlation coefficient was 0.89. Consistent with the computer vision literature we therefore found that our automatic algorithms perform as good as experts, with the added advantage of having per-frame analysis. To further support the use of automatic behavioral coding tools, in ref. ^9^ we showed that the automatic coding of head-turn to name-calling is more reliable than the corresponding question in the standard ADOS.

Apple’s open-source ResearchKit framework gave us the opportunity to extend our work beyond the clinical setting to reach caregivers and children in their homes. Our study, Autism & Beyond (https://autismandbeyond.researchkit.duke.edu/), is an iPhone-based app for caregivers and their children who are 12–72 months old. Using ResearchKit, an entire study—from a user-driven self-consent process to stimuli presentation, data collection, and user feedback—is integrated within a user-friendly app downloaded from the Apple App store.

**Fig. 1:**
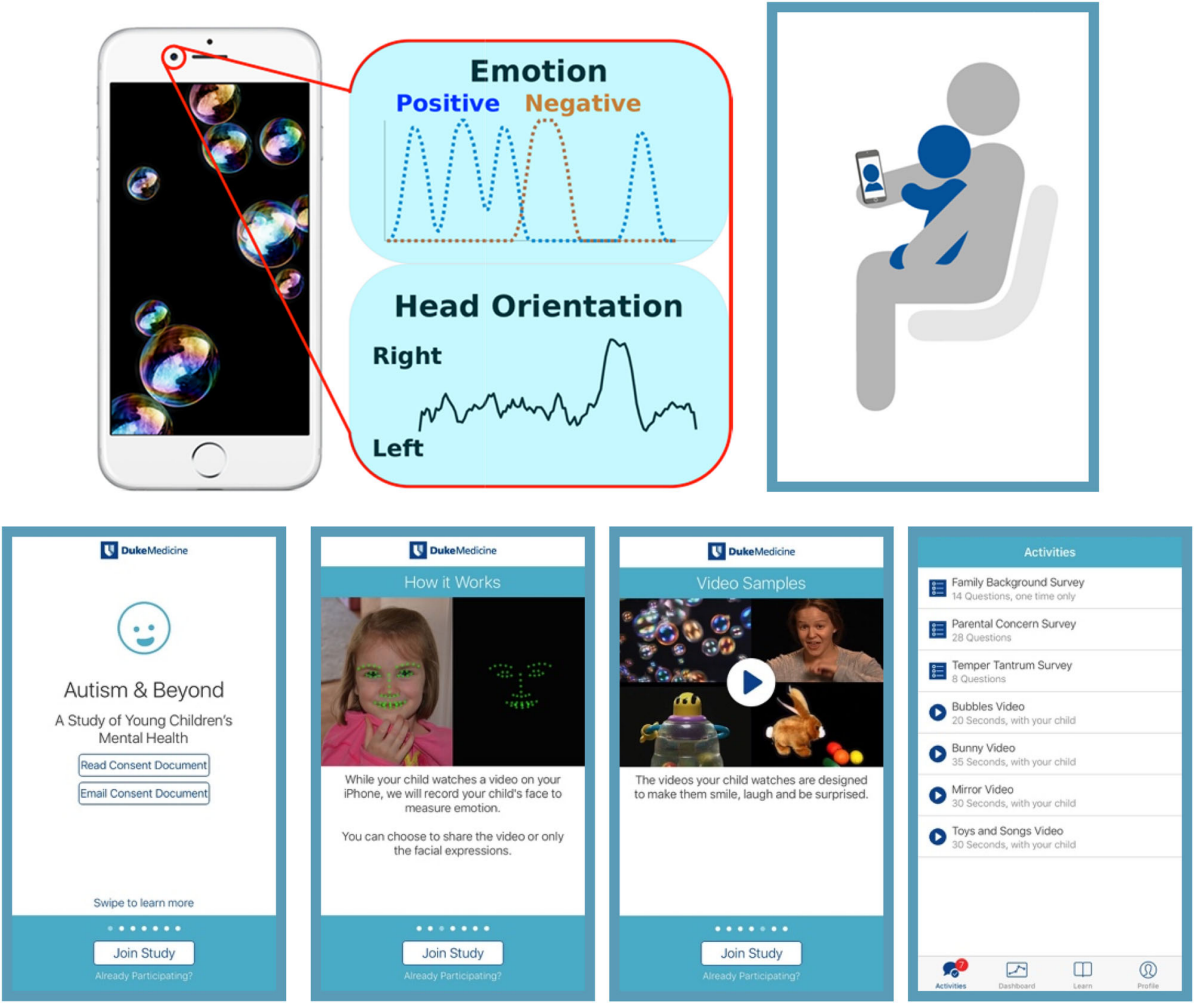
Autism & Beyond App. While children are watching neuroscience-based and clinically informed stimuli (i.e., short movies) on the iPhone’s screen, the iPhone’s camera records their facial/head behavior which is then analyzed either in the phone or after data is uploaded. All needed information integrated is integrated into the app, from e-Consent to questionnaires to the stimuli and the recording and (partial) analysis. Feedback information from the surveys is provided to the parents/caregivers as well. A few screenshots of the app are provided, illustrating the careful design to make it not only scientifically and medically relevant but also appealing and family friendly. All children and adults appearing in the app, e.g., to demo or to describe the study have given consent

The app functions as follows (Fig. 1 and S1). The caregiver downloads the app on his/her iPhone. After presenting on-boarding screens describing the study, the app guides the caregiver through an e-Consent process. If the caregiver meets inclusion criteria and provides consent, s/he then completes three to four brief questionnaires and presents four clinically informed stimuli (short movies, Video S1) to the child on an iPhone (or iPad). The camera on the device records a video of the child’s face as s/he watches the stimuli. Caregivers can choose to upload whole videos of their child or only the facial landmarks extracted using embedded face detection software encoding^15^ (http://www.humansensing.cs.cmu.edu/intraface/); see Video S2. The full videos of the child were uploaded to Duke Health servers and then emotions and attention are automatically encoded.^10^ The movies were revised versions of the ones in our iPad study and based on previous research described in refs. ^16–19^

The primary aims of the Autism & Beyond study were to test the acceptability and feasibility of conducting an iPhone-based study with caregivers and young children which included collection of caregiver-report and child video behavioral data and to test a new video-based approach for automatically collecting and quantifying young children’s emotions and behaviors in their natural environments. A secondary aim was to examine associations of the automatically coded emotions and behaviors with age, sex, and autism risk status. Answering these questions is the critical next step toward our goal of building an integrated digital platform to develop, pilot, and deploy mobile tools that use the capabilities of smartphones, carefully designed stimuli, and automated computer vision and machine learning analytics for screening and monitoring of young children’s autism risk, and eventually their broad cognitive and social-emotional development, in their homes, as well as clinical settings.

## Results

### Participation, recruitment, and enrollment

Figure S1 provides a flow diagram of caregivers’ engagement with the study. The study cohort includes participants who provided consent and completed all or part of the demographic survey including child’s age, providing therefore minimal information and use of the app (beyond just downloading it).

Figure S2 details enrollment across the months of data collection. Participants learned about the study through multiple sources, mostly social media, as indicated in the figure. Participants completed an in-app e-Consent process before enrolling; see Fig. S3 for examples of the e-Consent screens.

Table 1 presents the demographic characteristics of the children and caregivers in the final study cohort. Over 1 year, 1756 families with children aged 12–72 months old participated in the study (total number of downloads providing valuable information was 13,889), completing 5618 caregiver-reported surveys and uploading 4441 videos recorded in the child’s natural settings.

**Table 1:** Demographic characteristics

	*N* (%)
Child characteristics	
Total participants	1756
Sex^a^	
Boys	1211 (69.0%)
Girls	543 (31.0%)
Mean age in months (SD)^b^	40.4 (SD 16.3) (16.3%)
Race/ethnicity^a^	
Caucasian/not Hispanic or Latino	1120 (63.9%)
Caucasian/Hispanic or Latino	91 (5.2%)
African American	52 (3.0%)
Asian	76 (4.3%)
Multiple responses	453 (23.6%)
*Caregiver characteristics*	
Relationship to the child	
Parent	1716 (97.8%)
Other caregiver	39 (2.2%)
Sex^a^	
Female	1344 (76.5%)
Male	408 (23.2%)
Education	
Some high school	42 (2.4%)
High school diploma/GED	169 (9.7%)
Some college	439 (25.1%)
College degree	710 (40.5%)
Master’s degree	303 (17.3%)
Doctoral degree	88 (5.0%)
Employment^a^	
Employed out of home	1147 (65.5%)
Not employed outside of home	605 (34.5%)
Relationship status	
Single, never married	188 (10.7%)
Divorced or Separated	96 (5.5%)
Married or domestic partner	1447 (82.6%)
Widowed	9 (0.5%)
Other	12 (0.7%)
Number of children in the home (range 0–12 children)^c^	
1	539 (30.9%)
2 or more	1208 (69.1)
English primary language spoken in home	1590 (88.9%)

*Note*: Demographic characteristics of the children and caregivers in the final study cohort

^a^# Missing: child sex = 2; child race/ethnicity = 4; respondent sex = 4; employment = 4; children in the home = 9

^b^No significant difference of age by sex (mean age in mos: girls 39.0 (SD 16.3); boys 41.0 (SD 16.3) *p* = 0.9)

^c^Mean # of children in home = 2.2 (SD 1.2)

### Autism spectrum (AS) risk status of children

Two variables in the app measured the child’s autism risk status in the study: (1) caregiver-reported ASD diagnosis, and/or (2) a clinically significant score (>2) on the standard screening questionnaire MCHAT-R/F (here and in the tables called M-CHAT for brevity and consistency with the common language in clinics, though the follow-up questions were included in the app). The M-CHAT (see “Discussion” section where we address the differences between screening and diagnosis) was administered to the subset of caregivers of children aged 16–30 months old. A composite autism risk variable (autism risk, AS, to distinguish from ASD = autism spectrum disorder) included children who had a caregiver-reported autism diagnosis and/or a M-CHAT score indicating high risk status. Table 2 presents data on the autism risk status of the children in the study, overall and by age and sex.

**Table 2:** Autism risk status in cohort

Composite	*N* (%)	Mean age (SD)	*p-*value	Boys *N* (%)	Girls *N* (%)	*p-*value
Autism high risk	555 (31.6%)	43.6 (SD 15.6)	0.07	447 (36.9%)	108 (19.9%)	<.0001
Not autism high risk		39.3 (SD 16.6)		764 (63.1%)	435 (80.5%)	
Caregiver-reported ASD						
Caregiver-reported ASD	435 (24.8%)	47.9 mos (SD 13.3)	<.0001	354 (81.4%)	81 (18.6%)	<.0001
Caregiver did not report ASD	1321 (75.2%)	37.9 mos (SD 16.5)		857 (35.0%)	462 (35.0%)	
M-CHAT						
M-CHAT eligible	479 (27.3%)					
Completed MCHAT	407 (85.0%)					
M-CHAT high score	159 (39.1%)	24.1 (SD 4.1)	0.3	124 (44.0%)	35 (28.2%)	0.003
M-CHAT low score	248 (60.9%)	23.2 (SD 4.4)		158 (56.0%)	89 (71.8%)	

*Note*: Autism risk status in sample, overall and by age and sex. ASD stands for autism spectrum disorder and M-CHAT for the modified checklist for autism and toddlers-revised

#### Overlap of autism risk variables

Three hundred ninety-six children had only caregiver-reported autism diagnoses; 120 children had high-risk M-CHAT score and no caregiver-reported autism, and 39 children had both. Of children with a high M-CHAT score, 75% (*n* = 120) of caregivers did not report that their child had an autism diagnosis. Of the children with a low M-CHAT score, only 2.4% (*n* = 9) of caregivers reported an autism diagnosis.

There was no significant difference in the relationship between M-CHAT score and caregiver-reported ASD by age, by sex, or their interactions.

In the M-CHAT high group who did not have a caregiver-reported ASD diagnosis, one child was reported to have attention deficit hyperactive disorder (ADHD), but no other disorders. In the M-CHAT low group, three children who scored low on the M-CHAT had caregiver-reported diagnoses of developmental delay and “other” disorder, “other” disorder, and developmental delay.

### Activities performed

Screenshots of the activities screen are shown in Fig. 1 and S4. Table 3 (3a and 3b) provides details about the surveys and movies (each survey and each movie is a “task” or “activity”, surveys for caregivers to complete and movies for the children to watch), the number of movies viewed, and whether the videos could be analyzed using our automatic video coding, for the whole study cohort and for the M-CHAT sub-cohort.

**Table 3:** App activities

(a) Caregiver-report surveys		
Survey	Length	*N* (%)
Family background	14 questions	1756 (100%)
Parental concerns	28 questions	1692 (96.4%)
Duke temper tantrum screen	8 questions	1663 (94.7%)
MCHAT	~20 min	407 (85.0% of eligible)
Overall		5618 (97.8%)

(b) Child movie stimuli tasks								
	Whole cohort (*n* = 1756)				M-CHAT cohort (*n* = 407)^a^			
	All video	Full video		Landmarks	All video	Full	Usable	Landmarks
Video clip (duration)	*N* (%)	*N* (%)	Usable *N* (%)	*N* (%)	*N* (%)	*N* (%)	*N* (%)	*N* (%)
Bubbles (20 s)	1356 (77.2)	876 (77.2)	801 (91.4)	480 (77.3)	320 (79.0)	207 (51.1)	185 (89.4)	113 (27.9)
Bunny (35 s)	1084 (61.7)	698 (61.5)	618 (88.5)	386 (62.2)	257 (63.5)	168 (41.5)	147 (87.5)	89 (22.0)
Mirror (30 s)	1009 (57.5)	632 (55.7)	535 (84.7)	377 (60.7)	243 (60.0)	153 (37.8)	126 (82.4)	90 (22.2)
Toys and songs (30 s)	992 (56.5)	619 (54.5)	521 (84.2)	373 (60.1)	238 (58.8)	154 (38.0)	127 (82.5)	84 (20.7)
Total (115 s)	4441 (63.2)	2825 (62.2)	2475 (87.6)	1616 (65.1)	1058 (65.3)	682 (42.1)	585 (85.8)	376 (23.2)

^a^Percentages represent proportion of M-CHAT eligible sample

#### Surveys

Overall, caregivers completed a mean of 3.2 (SD 0.6) out of 4 possible caregiver-reported surveys. For the three surveys for which all subjects were eligible (i.e., family background, parental concerns, and temper tantrums; see Supplementary Material), 1645 (93.7%) caregivers completed all three surveys, 65 (3.7%) completed two surveys, and 46 (2.6%) completed one survey, 407 (85% of eligible) caregivers completed the M-CHAT. The caregivers of older children who were not eligible to complete the M-CHAT (*n* = 1277) completed a mean of 2.93 surveys (SD 0.33) with 1221 (95.6%) completing all three surveys, 26 (2.04%) completing two surveys, and 30 (2.35%) completing one survey. The caregivers of M-CHAT eligible children completed a mean of 3.7 (SD 0.74) surveys (399 (83.3%) completed four surveys, 33 (6.89%) completed three, 31 (6.47%) completed two, and 16 (3.34%) completed one survey). Caregivers of children in the high autism (AS) risk category completed significantly more surveys than children in the low-risk category (high risk 3.3 surveys; low risk 3.1 surveys; *F* = 43.02, *p* < 0.0001). There was no significant difference in number of surveys completed by child sex.

#### Overall number of movies viewed

The mean number of movies viewed was 2.5 (SD 1.6). Overall, 809 children (46.1%) viewed four movies, 166 (9.5%) viewed three, 275 (15.7%) viewed two movies, 157 (8.9%) viewed one movie, 349 (19.9%) viewed no movies. There were no significant differences in movie completion by child’s sex, age, or autism (AS) risk status.

#### Videos—full video vs. landmarks sharing

**Fig. 2:**
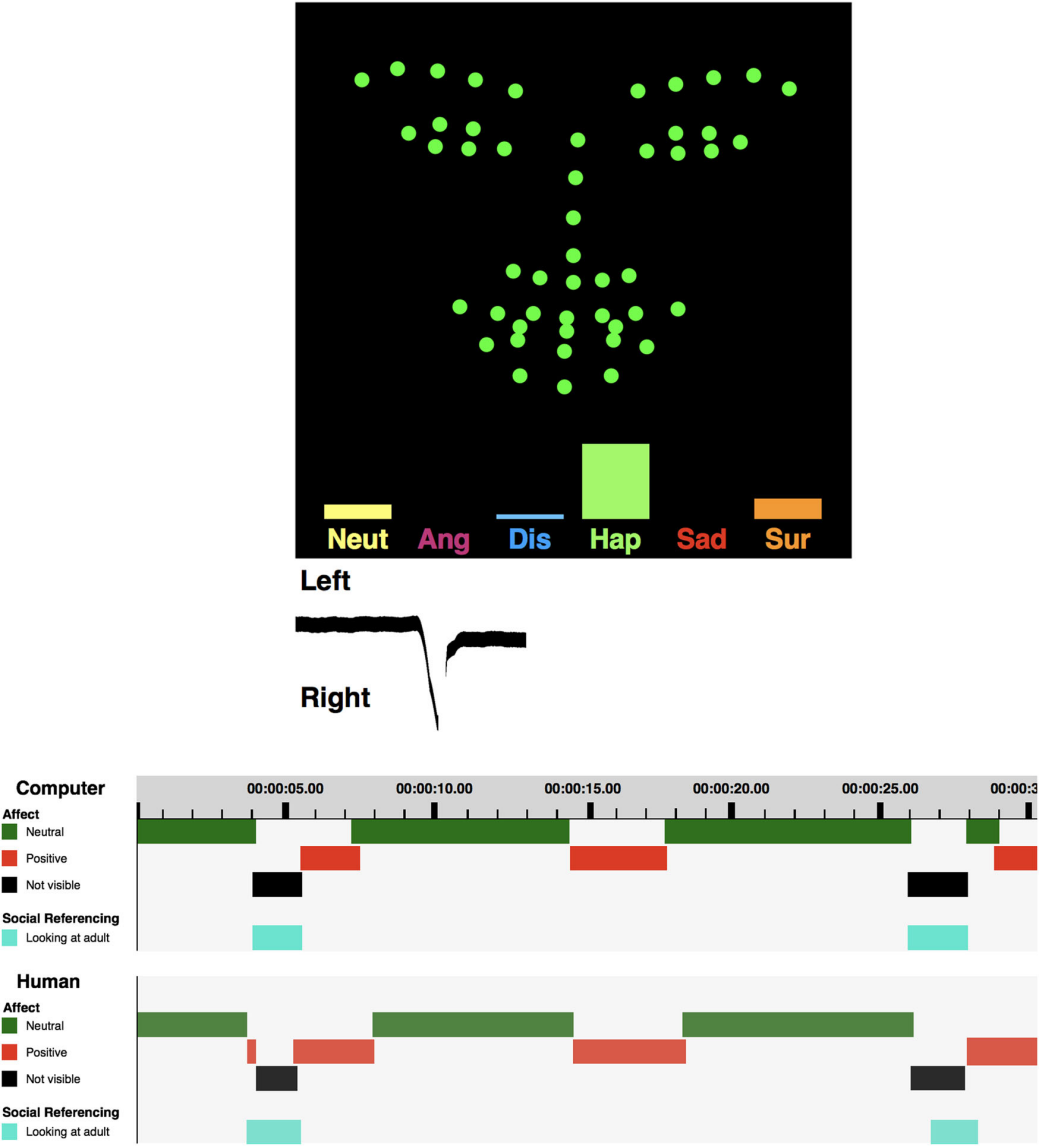
Automatic coding and validation. The algorithm automatically encodes, from detected features, as marked in the figure (top), both the head position and the emotion while the child is watching clinically informed movies. From these we can infer their attention, social referencing, and emotional response to the stimuli. The automatic coding has been carefully validated (bottom, timeline of emotions comparing manual and computer coding)^10^

One thousand one hundred and thirty-five caregivers (64.6%) agreed to upload full videos of their children. Six hundred and twenty-one caregivers (35.4%) chose to upload only facial landmarks from the videos (see Table 3b, Fig. 2, and Video S2). Caregivers of children in the high-risk AS group were significantly more likely to agree to share the full video (69.6 vs. 62.4%, *F* = 8.54, *p* = 0.004). There were not significant differences by sex or age.

#### Uploaded full videos

We conducted computer vision analyses on the full videos uploaded. Of the 1135 caregivers who agreed to upload whole videos, 903 (79.6%) watched at least one movie (mean 2.5; SD 1.6), 508 (44.8%) watched four movies, 105 (9.3%) watched three, 188 (16.6%) watched two movies, 102 (9.0%) watched one movie, and 232 (20.4%) watched no movies.

Overall, 2475 (87.6%) of the videos collected could be analyzed using our computer vision algorithms. Table 3b provides rates of usable data for each of the videos. There was no significant difference by sex, age, or autism (AS) risk status between usable and not usable videos.

### Automatically quantified emotions and attention

For each video, we automatically quantified the percentage of positive emotion, negative emotion, and neutral expression, as well as the child’s attention (see Fig. 2 and Video S2). Details on how these are computed are presented in the Supplemental Information and in ref. ^10^ We examined whether there were significant associations between coded emotion and attention variables and child age, child sex, and AS risk, for the whole sample and for the M-CHAT sub-sample.

#### Child age

**Fig. 3:**
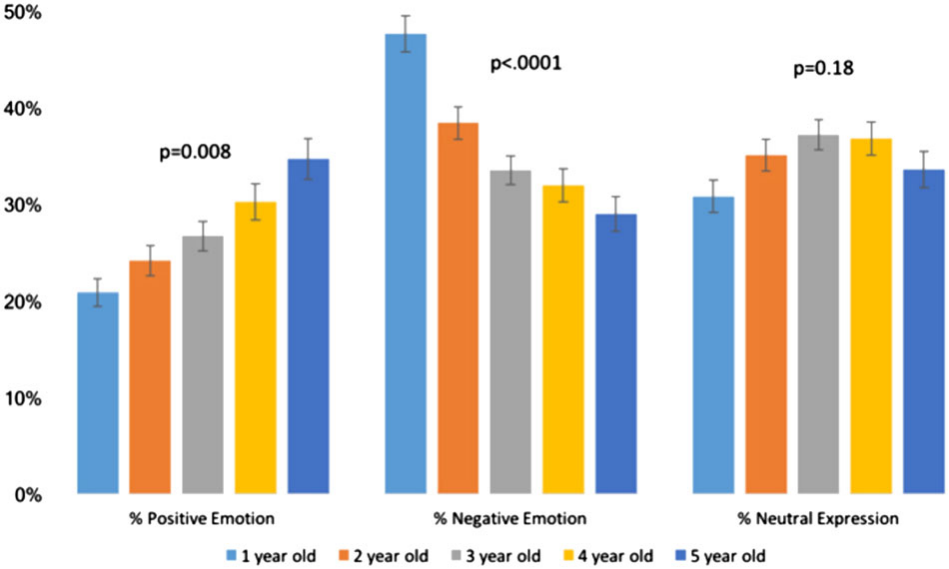
Age and emotions association. Associations between age and mean percentage emotions across all four movie tasks in the whole cohort (*n* = 1756)

With increased age, children showed a greater percentage of positive emotion, a lower percentage of negative emotions, and no significant differences in the percentage of neutral expression across all videos (Pearson Correlation Coefficients with age: total positive emotion 0.208, *p* < .0001; total negative emotion −0.262, *p* < .0001; total neutral emotion 0.047, *p* = 0.17). Figure 3 illustrates these relationships. The only significant difference of attention by age was found for the bubbles movie with older children (36–72 months) attending a mean of 84.5% of the time and younger children (12–35 months) attending a mean of 78.5% of the time (*p* = 0.02).

#### Child sex

Because of the preponderance of boys in the sample and the higher rate of autism risk in the boys, we did not separately examine the relationship between the video variables and child sex. Interactions with sex and autism risk are noted below.

#### AS risk: whole cohort

Table 4 reports the mean percent emotion and standard error by movie task and AS risk status based on caregiver report and/or M-CHAT score, overall and by sex, adjusted for child age in months.

**Table 4:** Mean percent emotion (standard error) by video clip and autism spectrum risk status based on caregiver report and/or M-CHAT score, overall and by sex, adjusted for child age in months

Video clip	*N*	All children					*N*	Boys					*N*	Girls				
		High risk		Low risk				High risk		Low risk				High risk		Low risk		
		Mean	SE	Mean	SE	*p*-value		Mean	SE	Mean	SE	*p*-value		Mean	SE	Mean	SE	*p*-value
Neutral emotion																		
Bubbles	781	41.2	1.8	35.8	1.2	**0.01**	550	39.8	1.8	32.9	1.5	**0.004**	231	39.7	3.7	39.2	2.0	0.89
Bunny	608	39.2	1.9	33.1	1.3	**0.01**	433	38.0	1.9	31.9	1.6	0.01	175	40.4	4.0	34.3	2.1	0.18
Mirror	530	36.2	1.8	31.4	1.2	**0.02**	380	36.1	1.7	30.5	1.5	**0.01**	150	34.2	4.1	32.6	2.0	0.72
Toys and songs	509	41.1	2.3	34.6	1.5	0.64	365	37.7	2.2	35.3	1.9	0.39	144	41.2	5.5	44.8	2.7	0.56
Positive emotion																		
Bubbles	781	24.7	1.8	27.0	1.2	0.26	550	25.9	1.9	28.8	1.5	0.23	231	24.7	3.4	25.0	1.8	0.95
Bunny	608	24.9	1.9	26.5	1.3	0.45	433	24.6	1.9	27.5	1.6	0.24	175	27.8	3.8	24.9	2.1	0.51
Mirror	530	29.9	1.9	35.1	1.3	**0.02**	380	28.2	1.9	34.2	1.6	**0.01**	150	33.7	4.4	35.7	2.2	0.68
Toys and songs	509	23.3	2.1	25.6	1.5	0.34	365	24.4	2.1	27.8	1.8	0.22	144	24.5	4.8	23.1	2.4	0.79
Negative emotion																		
Bubbles	781	34.1	1.7	37.1	1.2	0.13	550	34.3	1.8	38.3	1.5	0.08	231	35.6	3.7	35.9	2.0	0.94
Bunny	608	35.9	1.9	40.4	1.4	0.04	433	37.4	1.9	40.6	1.6	0.20	175	31.9	4.1	40.8	2.2	0.06
Mirror	530	33.9	1.7	33.5	1.2	0.83	380	35.7	1.7	35.3	1.5	0.85	150	32.1	3.8	31.7	1.9	0.92
Toys and songs	509	35.7	2.1	34.6	1.5	0.64	365	37.9	2.1	37.0	1.8	0.73	144	34.3	4.9	32.1	2.5	0.70
Attention																		
Bubbles	800	90.0	1.4	92.7	0.9	0.08	563	90.4	1.4	91.7	1.2	0.49	237	86.4	2.8	94.3	1.5	**0.01**
Bunny	617	89.0	1.7	88.9	1.2	0.94	440	89.7	1.7	87.0	1.4	0.23	177	83.0	3.6	91.7	1.9	0.03
Mirror	534	86.3	1.7	87.4	1.1	0.56	383	87.1	1.6	85.6	1.4	0.50	151	78.8	3.8	90.1	1.9	**0.01**
Toys and songs	521	92.3	1.9	91.3	1.3	0.65	374	90.3	2.0	88.3	1.7	0.44	147	91.6	3.4	94.7	1.7	0.41

*Note*: Bolded *p-*values remains significant after performing the Benjamini–Hochberg procedure,^44^ with a 10% false discovery rate to account for the impact of multiple comparisons

#### Differences in neutral emotions

As shown in Table 4, overall children with high AS risk had a significantly increased percentage of neutral emotions compared to low-risk children while watching the bubbles, bunny, and mirror stimuli. Boys with high autism risk showed a significant increased percentage of neutral emotions with the bubbles and mirror movies. These *p-*values remained significant after correcting for multiple comparisons.

#### Differences in positive emotions

As shown in Table 4, in the mirror movie, lower mean percentage of positive emotions was significantly associated with high autism (AS) risk status for all children and boys in particular. These values remained significant after correcting for multiple comparisons.

#### Differences in negative emotions

After correcting for multiple comparisons, we did not find significant associations between autism risk status and percentage of negative emotions.

#### Differences in attention

We found no significant differences for attention for any of the movies when controlling for age and sex. When we stratified by sex and adjusted for age, we found that girls but not boys with high AR had significantly lower mean percentage of attention than girls with low AR for the bubbles, bunny, and mirror videos. The association with the bubbles and mirror video remained significant after correcting for multiple comparisons.

#### AS risk: M-CHAT sub-cohort

Of the 407 caregivers who completed the M-CHAT, 334 (82.1%) of their children watched at least one movie (Table 3b). Neither emotion nor attention showed significant differences in the association with low and high-risk M-CHAT groups.

As shown in Table S1, we found significant difference in mean percentage of positive emotions for children with a high M-CHAT score (8 or more) showing lower percentage of positive emotion while watching the bubbles movie compared with medium and low-risk children. This association was only significant for boys. However, these *p-*values did not remain significant after correcting for multiple comparisons.

We then examined whether the video extracted emotion and attention variables predicted M-CHAT continuous score controlling for age and sex. With increasing M-CHAT score (i.e., increasing ASD risk), the percentage of negative emotion in the toys and songs video increased (*p* = 0.003, *F* = 8.96); the percentage of neutral emotion increased in the bubbles (*p* = 0.0002, *F* = 14.30), bunny (*p* = 0.0036, *F* = 8.74), and mirror videos (*p* = 0.0004, *F* = 13.46); and across all four videos, the percentage of positive emotion decreased (bubbles: *p* < .0001, *F* = 19.2; bunny: *p* = 0.001, *F* = 10.67; mirror: *p* < .0001, *F* = 17.82; songs/toys: *p* = 0.01, *F* = 6.8).

No difference in attention in relationship to categorical or continuous M-CHAT score was found.

## Discussion

### Feasibility

Previous studies of home videos of infants and toddlers have used human coding to demonstrate differences in attention and affect that distinguish children with autism and typical development vs. developmental delay without autism.^20–23^ This study extends previous work in a number of ways, including (1) using a smartphone to collect data in children’s natural environments (e.g., homes), with a full study integrated in one device; (2) engaging caregivers directly in the collection of video data of young children using developmentally sensitive video stimuli; and (3) conducting a national (and currently extended internationally^24^) population study solely through an app available on the Apple App Store.

Our results demonstrate that it is feasible to conduct child development research studies with caregivers and young children in their homes using traditional caregiver-report surveys and new video-based behavioral assessment. Our enrollment in this study was ten times greater than in our clinic-based study during a similar time frame.^8^ While our enrollment spiked after Autism & Beyond was highlighted during Apple’s press releases and at Apple’s World Wide Web Conference, caregivers continued to enroll in the study throughout the year. More than half of caregivers learned about the study through social media, a finding that suggests that recruitment for all research studies should leverage new ways to reach potential participants, particularly young participants. We also found that two-third of caregivers were willing to upload the full video of their children while a third opted to upload the extracted facial landmarks. As we are developing the computer vision algorithms, full videos give us the opportunity to test and refine these algorithms. Our long-term goal is to be able to conduct the full coding of the videos on the phone so that the videos of children not need to be uploaded, improving the children’s privacy.

With the vast majority of the videos being usable for automatic coding, we learned that it is feasible to collect high-quality video using a smartphone camera with the video collected by the participants without in-person guidance. In previous studies, including our own, trained assistants obtained videos of the children viewing the stimuli. Here, caregivers followed simple directions presented pictorially and recorded the videos in their homes with no additional instruction. Our finding that there were no significant differences by sex, age, or autism (AS) risk status between usable and not usable videos shows that this approach can be used with very young children (1–6 years old in this study) and children at high risk for autism, both populations who commonly present challenges for observational data collections in standard research and clinical settings.

Our data also provided us with preliminary insights about the acceptability of stimuli presented in the movie clips, some of which have been used in previous autism studies.^18,19^ The order of the movie list on the app corresponded with the response rates: the greatest number of participants completing the movie at the top of the list (bubbles) and the least number of caregivers completing the last movie (songs/toys) in the list. Future studies should randomize the order of the movie presentations to reduce risks of an order effect. A key part of our ongoing program of research is to create and test movie stimuli for their capacity to tap into key constructs being studied, their acceptability to children, and their developmental appropriateness. Here, we used the same movies for the whole sample. Future studies should develop and deploy movie stimuli targeted to developmental age, as well as autism (AS) risk status.

### Relationship of automatically coded emotion and attention with child sex, age, and autism risk status

We did find relationships between emotions and attention and age, sex, and autism risk status. These findings in this convenience sample gives us insight into whether our movie tasks and automatic coding of emotion and attention are aligned with current clinical literature on young children overall and children at high risk for autism specifically. First, across all of the videos, percentage of positive emotions increased and negative emotions decreased with age. There was no difference in neutral emotion. These data may reflect that our stimuli, across the board, were perceived as more emotionally engaging by the older children compared to the younger ones. However, degree of attention to the stimuli was similar across age. We also find sex differences. However, our higher representation of boys and of male children with autism means that our sample does not represent population-level differences by child sex so we only reported child sex differences by autism (AS) risk status.

We found significant differences in automatically coded emotions by autism risk status controlling for age and sex. First, across all of the videos, except the toys/songs stimuli, children in the whole cohort high autism risk group showed increased neutral emotion compared to children not in the high-risk group. This finding is consistent with previous research showing that children with autism have decreased engagement and decreased range of emotion.^5,17,25–27^ With the mirror stimulus specifically, children in the high autism risk group showed decreased positive emotion. We cannot say whether this is related to viewing of faces overall or is specific to looking at themselves. We do have a hint that it may be specific to looking at their own image because we do not find any significant differences by autism risk when the children observed the social sections of the songs/toys video. When stratified by sex, we found that the differences in neutral emotions for high-risk children were specific to boys.

In the smaller M-CHAT cohort where autism risk was assessed with a validated measure, we found that the children with high-risk score (8 or more) compared to those with a low risk (2 or less) had lower percentage of positive emotion in the bubbles video. When we examined M-CHAT as a continuous variable, we found increases in percentage of negative and neutral emotions and decreases in positive emotion, findings which are consistent with previous studies,^5,16,28–34^ and with the result for the whole sample. However, our M-CHAT findings did not remain significant after correcting for multiple comparisons.

Our finding of a trend of decreased attention for the high autism group in the whole cohort (and not the M-CHAT sub-cohort) with the bubbles stimulus is consistent with previous studies.^35^ When stratifying by sex, it was girls, not boys, at high autism risk who had decreased attention compared to low autism risk girls with the bubbles and mirror videos. These differences remain significant after correction for multiple comparisons. The differences in attention by sex and autism risk is intriguing in light of growing research examining the differences in autism presentation and identification for boys and girls.^36–39^ Though non-conclusive, our data suggest that our approach can be used to explore potential sex differences in autism risk.

It is possible that the finding that differences in responses to the videos were sometimes more robust for boys is due to the different sample sizes for males vs. females and inadequate power to detect group differences for girls. Alternatively, recent studies have shown that there exist sex differences in patterns of symptom expression among children diagnosed with autism.^40^ Larger samples will help to further explore this.

### Screening, diagnosis, and tracking

The future goal of automatic tools as those here described is to improve the accuracy of, as well as access to, screening. More accurate screening is needed given the high rate of false positives associated with the M-CHAT and would allow resources to be used more efficiently, e.g., by reducing or prioritizing wait lists. Symptoms of autism tend to worsen from 12 to 24 months and monitoring this developmental trajectory with low-cost and easy access tools like this app could improve the accuracy of the screening tool. Such tools could also provide valuable longitudinal and objective behavioral information to clinicians involved in diagnosis and treatment.^3,4^ This could provide clinicians with a daily picture of progress, allowing real-time treatment adaptation. This is in sharp contrast with current practices of feedback provided to clinicians during sporadic visits. Finally, similar tools could potentially be developed to train therapists, and observe and quantify their behavior during therapy delivery.

### Study limitations

While a clear demonstration of the feasibility of video-based behavioral coding at home, our study has a number of limitations. A detailed discussion of these limitations and how to address them is provided in the Supplemental Information. These limitations include participants’ non-uniform representation of the general population, assessment of autism risk based only on M-CHAT and caregiver-report (contrary to our other work in the clinic^9^), the need for larger clinical validation studies which also address specificity and co-morbidity, the need to observe and automatically code other people present during the study in order to eliminate potential co-founding variables, and limitation of the study to just iOS devices. The need to add complementary behavioral markers, e.g., language and repetitive behaviors, is addressed in the Supplemental Information as well.

### Study ethical issues

We are committed to defining the ethics of digital health research and population-level big data, particularly with vulnerable populations, which include children and people with developmental disability and mental illness. A full discussion of ethical issues common to mobile health and how we carefully handled them in the Autism & Beyond design is provided in the Supplemental Information. They include e-Consent and self-assessment of eligibility, privacy and right to withdraw, and clinical follow-up when risk for ASD is detected. Our team had an ethics consultant for this study, and we believe that with proper mitigation and high standards, the potential benefits and ethical considerations of helping populations in need motivate the development of complementary mobile health tools as the one here described.

### Next steps

We will need to further validate the reliability, validity, specificity, and utility of our tools in representative populations of children and then replicate these findings in independent samples. The design of these studies is relatively straightforward, in particular studies that compare digital tools to standard clinical evaluation approaches.^8–10,24^ Children with a large diversity of developmental disorders need to be included in the study to further understand both the feasibility of the approach and its sensitivity/specificity, including handling co-morbidity. Furthermore, the willingness of the professionals to use the outcomes of mobile behavioral apps needs to be investigated. The more accurate and properly validated the results are, the easier the path to adoptions becomes. For example, in our earlier study of digital adaptation of the M-CHAT,^8^ the positive impact on accuracy of screening and appropriate referral was so strong and the device so easy to use that clinicians requested to keep the tablet for regular clinical use even after the study was completed. We also believe that our program of research to develop new tools needs to be linked to the creation, testing, deployment, and evaluation of guided advice, coordination with healthcare providers, and even interventions to improve the lives of the children and families who participate in studies.

## Conclusions

While the tools we describe here are for research, they are the foundation for the development of clinically informed screening tools to provide caregivers and clinicians accessible, affordable, and scalable tools for early identification and symptoms monitoring of autism and other developmental and mental health challenges. With this study, we have demonstrated the feasibility of this approach: caregivers are willing to participate; the survey and video data they collected themselves in their homes is high quality; we are able to apply computer vision algorithms to the video data and quantify the observed behavior of young children; and the association of the automatically coded behaviors with age and autism risk are consistent with research conducted in traditional research settings. While the work here described concentrated on stimuli and coding related to autism, these positive feasibility results open the door to investigate other developmental and mental health challenges that require observing behavior. These include for example ADHD, where measuring attention is critical and possible (including gaze^41^), and post-traumatic stress disorder, where movie stimuli can be designed to elicit emotions which can be efficiently measured via both face emotion (as in this work) and heart rate analysis.^42^ Extending the work to other disorders entails designing new stimuli that will elicit different symptoms associated with those conditions, accompanied by the corresponding automatic coding.

It is noteworthy that results of our earlier studies and this one, which used similar stimuli^9,10^ were similar, suggesting that data collected in the clinic and in the natural environment (via Autism & Beyond) will be comparable. This is a positive first step indicating that the behavioral stimuli designed for clinical environments can be translated to home analysis and vice versa.

Smartphone-based community-level research enables us to reach children and families who are often excluded from participating in research. Feasible, low-cost, and scalable tools to enable caregivers to obtain information about their children’s development in the home both increase the ecological validity of the data collected and increase caregivers’ access to knowledge about their child’s autism risk. Linking results from these tools with pediatricians and developmental specialists will also help caregivers to overcome the current barriers to early identification and intervention. Increased access to knowledge, we hope, will also increase public awareness about autism risk and lead to greater access to care. Eventually, effective guided advice and interventions may be administered through these same technologies.

We envision the development of a digital platform that will be globally accessible, affordable, and easy to use with automated, time-efficient, and individualized methods for analysis and interpretation of young children’s emotions and behaviors.^43^ The population-level and individual child data that we will be able to collect with this platform will enable us to improve the translation of science into practice and give many more families access to evidence-based knowledge that can help them to support their children’s development and mental health.

## Methods

Details on the app design strategy and software are provided in the Supplemental Information.

### Data availability

Please contact guillermo.sapiro@duke.edu or geraldine.dawson@duke.edu to obtain access to data and code for research-purposes only. This includes both codes for reproducing the tables and for building basic app blocks (components also shared via github). Analyzed data is also available from the authors. Raw data (videos) cannot be shared due to privacy protection.

## Electronic supplementary material

Supplemental Material(PDF 1433 kb)

Video S1

Video S2

## Electronic supplementary material

**Supplementary information** accompanies the paper on the *npj Digital Medicine* website (10.1038/s41746-018-0024-6).
