# Supplementary material for: Automatic emotion and attention analysis of young children at home: a ResearchKit autism feasibility study
Source: NPJ Digit Med. 2018 Jun 1;1:20. doi: 10.1038/s41746-018-0024-6 (PMC6550157; doi:10.1038/s41746-018-0024-6)
Supplement: Supplementary file 1 — Supplemental Material(PDF 1433 kb) [file 41746_2018_24_MOESM1_ESM.pdf]

Supplemental Information

Automatic Emotion and Attention Analysis of Young Children at Home: A ResearchKit Autism Feasibility Study

Helen L. Egger, Geraldine Dawson, Jordan Hashemi, Kimberly L. H. Carpenter, Steven Espinosa, Kathleen Campbell, Samuel Brotkin, Jana Shaich-Borg, Qiang Qiu, Mariano Tepper, Jeffrey P. Baker, Richard A. Bloomfield, Jr. and Guillermo Sapiro

Details on app design, content, and data analysis

Apple iOS ResearchKit. Apple's iOS ResearchKit enables researchers to create mobile iOS applications to obtain consent and conduct medical research studies. Using the ResearchKit informed e-consent and survey modules and study-specific active tasks to collect video data, we created the first pediatric ResearchKit app: Autism & Beyond. This is also the first ResearchKit app to collect video of the participants. The Autism & Beyond app was made available on the US Apple App Store October 15th, 2015. We collected the data used in this paper for one year.

The team. The study was developed by an interdisciplinary team of pediatric mental health and development specialists (child psychiatrists and psychologists, pediatricians, neuroscientists, epidemiologists, global health researchers), engineers, data scientists, app developers, designers, IRB experts, back-end app management, and computer scientists at Duke University and Duke Health. Duke University and Duke Health did all the development internally.

Technical specifications. The app works on the iPhone 4S and above. It also works on equivalent iPad generations. The Autism & Beyond source code is available to the research community upon request. Groups interested in direct use of the app as it is for their study can also do so via collaboration with the Autism & Beyond team, due to privacy and consent considerations as well as backend designs, and the data will be stored in a separate partition at Duke Health, e.g., [Kumm, 2017]. Only data collected via the app is stored, with proper encryption and security approved by the Institutional Review Board. If the study includes other participants' data, it will be combined at the study site following their privacy and security protocols.

The app included a total of 3 versions: 1.01 was our initial launch; 1.02 included minor UI bug fixes, text updates, and a fix to preprocessing with video data; 1.03 included additional minor UI fixes and an update to the consent. None of the fixes affected the study (movie presentation and data collection). We also recorded over 95% of sessions without crashes.

Data storage. Data were stored at Duke Health behind the hospital's firewall. All data access by the team was done via virtual machines for privacy protection and security considerations.

Participant onboarding. After downloading the app, the prospective participant self-navigated through the descriptions of the study (see figures 1 and S3), eligibility criteria (included below) and then a self-guided e-consent process approved by the Duke University Medical Center IRB.

Study inclusion criteria. Inclusion criteria included: the adult participant was parent/legal guardian of child, the child was between 12 and 72 months old, the parent/guardian could read English, and the parent/guardian lived in the United States (the study was used in South Africa in a community setting as well [Kumm, 2017]). Caregivers with children with and without autism were eligible for the study.

The age range was selected to include M-CHAT applicability as well as when ASD diagnosis is possible. By collecting data on a large age range (12-72 months), we were able to examine whether there are important developmental differences in attention and interest in the videos and behavioral responses.

Informed consent. Informed consent was obtained through the app itself (see also Figure 1 and Figure S3 for some screenshots of information). Caregivers are led through a series of screens with information about the study, risks, and benefits. If they agreed to participate in the study and signed the consent with their finger on the screen, this signed copy of the e-consent was sent to their email address with a prompt to affirm that they want to participate in the study. Caregivers could not enroll in the study without responding affirmatively to the confirmation email.

Additional human subject protections. To ensure that caregivers were fully informed about the videos we were collecting of their child, we provided a thumbnail copy of the video (side by side with the movie stimulus clip) on the dashboard tab (see Figure S4) for the parent to review at any time. Note that this video thumbnail is not presented during the child participation, is

presented in the dashboard to the caregivers after the child has finished. During child participation the screen only shows the movie stimuli. If a parent withdrew from the study, we agreed to erase all data, including videos of their child. We also provide a dedicated email address and phone number for reaching the study team. We reviewed these emails and calls and provide responses where warranted. We also had a dedicated website [https://autismandbeyond.researchkit.duke.edu/].

Data sharing options. To participate in the study, caregivers had to agree to share either the full video of their child or facial landmarks extracted using IntraFace [IntraFace]; see Figure 2 and Video S2. Because of the sensitivity of video data of children, we did not include a choice, as other ResearchKit studies have [Bot, 2016; Webster, 2017], for sharing of data outside of our study team.

Basic app structure. Once enrolled in the study, participants had access to four tabs: activities, dashboard, learn, and profile (Figure S4).

- *Activities tab.* The activities tab provides a list of activities/tasks (Figure 1 and Figure S4), parent-report surveys and movie stimuli (Video S1) to be completed. Table 3 summarizes the activities. Participants could skip questions in the surveys. The four movie stimuli could be done in any order. Instructions were provided visually and succinctly.
- *Dashboard tab.* This includes the following content:
    1. Real time data about all participants (e.g., age distribution of children in the study)
    2. Progress/timeline for completion of tasks;
    3. Individualized feedback: thumbnails of video of child and stimulus side by side, results of temper tantrum survey, and results of the M-CHAT (if the child was eligible to complete). This is available to the caregivers following the completion of each corresponding activity. This follows standard clinical practice, e.g., caregivers get

information about the M-CHAT when completing it and before, e.g., doing an ADOS. This protocol, in contrast to waiting for the completion of all app activities before any feedback, might for example discourage caregivers from continuing with the other tasks. On the other hand, we wanted to give parents useful information even if they decide not to complete all the activities.

Some screen shots are provided in Figure S4.

- *Learn more tab.* This tab provides further information about the study, how this study works, who can participate, who is running this study, online resources, and some legal notices. Some screen shots are provided in Figure S5.
- *Profile tab.* The Profile tab contains everything for users to manage their accounts and device settings including: setting reminders, setting app permissions, changing their passcode for their study account, changing their video sharing options, signing out, reviewing their consent, reviewing the privacy policy, sending feedback, and withdrawing from the study.

Measures. See Table 3 for further details about measures.

Caregiver-report surveys. All participants were asked to complete three parent-report surveys: Family Background Survey; Parental Concerns Survey; and the Duke Temper Tantrum Screen [Egger, 2003]. These appear below. Results on the Parental Concerns Survey and Temper Tantrum Screen will be presented in future reports; in this feasibility study we concentrate on reporting measures related to ASD.

Caregivers with children ages 16 to 30 months were asked to complete the Modified Checklist for Autism in Toddlers-Revised with follow up questions incorporated in the survey (M-CHAT-R-F/U; here referred to as M-CHAT), a 20 question autism screening measure widely used in primary care [Robins, 2013]. The M-CHAT classifies children into three autism risk categories: high risk (score 8 or greater), medium risk (score of 3-7), and low risk (score of 0-2). The

creators recommend that if a child screens in the medium risk category (3-7), they also answer a secondary set of questions to clarify their responses to the initial set and which allows clinicians to further classify the result as high or low risk. In validation studies, approximately half of children scoring in the high risk category after screening with both the initial question and follow up questions went on to receive a diagnosis of autism [Chlebowski, 2013]. The digital version of the M-CHAT-R/F within the Autism & Beyond app presented all follow up questions for failed items, regardless of final score. Therefore, children were classified as low risk if answers indicated a persistent score of 0-2 and high risk if answers indicated a score of 3 or higher.

Online versions of the M-CHAT are available to families (https://www.m-chat.org/mchat.php) and several studies have created digital versions that caregivers can self-administer at home prior to their clinical visit [Brooks, 2016].

Movies Stimuli. We also asked caregivers to have their children watch four stimuli/movies. Children viewed four separate movie clips: bubbles, bunny, mirror, and toys/songs (see Video S1 for segments of the movies). These movies were adapted from our previous work conducted in the primary care setting [Campbell, 2016; Campbell, 2017; Campbell, 2017b] and designed to elicit autism symptoms, based on [Dawson, 2004; Jones, 2016; Jones, 2017]. As detailed before, automatic encoding of children's responses to watching these movies is the key innovation of this approach (Video S2).

Feedback. Caregivers who completed the M-CHAT received the M-CHAT score and an indication whether this score placed the child in the low risk, moderate risk, or high risk group. If children scored in the moderate or high risk category, the caregivers were provided an active link to Autism Speaks' map of the US with early childhood intervention locations in each state (https://www.autismspeaks.org/). Caregivers who completed the temper tantrums screen received feedback on their child's tantrums compared to other preschool children. Children with

frequent and aggressive tantrums were given links to parenting resources and possible mental health evaluation for their child. Feedback is stored under the dashboard tab. M-CHAT and temper tantrum feedback can be emailed or printed from the app as a PDF.

Caregivers did not receive feedback about the video data due to its research nature.

See Figure S5 for examples of the feedback provided to caregivers.

Video processing. In this paper we present video data from the videos uploaded to the server. We excluded the video landmark data since that option was only included in the app to learn about participants' willingness to be involved and the work with the uploaded full videos enabled the development and use of more advanced face analysis algorithms. We did conduct a manual review of each video to ensure that we had collected data on young children, not older children or adults.

To extract key variables, we first automatically detect 49 facial landmarks via IntraFace [IntraFace], which could be running on the iPhone itself. If the participant's caregivers upload the videos, an option provided in the app, additional analysis is possible via both updated versions of IntraFace and custom video analysis algorithms developed by the authors. The work here reports results on such uploaded videos, which also permit more careful validation.

Then, given these frame-by-frame facial landmarks, raw facial information such as head pose and emotion is automatically coded through machine learning and computer vision algorithms [Hashemi, 2015; Hashemi, 2015b; Intraface]. In addition, higher level facial information can be computed, such as head turns [Hashemi, 2015]. All this is done at the time resolution of the recorded video, 20-30 frames a second, which is significantly higher than the normal time resolution in human manual encoding. Examples of such automatic facial landmarks, emotions, and head position are provided in Figure 2 and Video S2.

For the purpose of the work here reported we focused on four variables extracted from the

recorded videos, namely: percentage of time paying attention to the visual stimuli on the iPhone screen, number of head turns, emotion profile, and yaw angle profile. We consider the participant looking at the screen when he/she exhibits a yaw pose magnitude that is less than 45° (this is again automatically computed from the landmarks). Then the percentage of time paying attention is computed by dividing the number of frames in the video where the participant is looking at the screen by the total number of frames in the video. A head turn is defined as a sequence when the face exhibits a yaw pose magnitude of less than 45°, moves past 45°, and then comes back to under 45°. Our head turn implementation automatically detects instances where the participant is not looking at the screen and the maximum yaw pose magnitudes of the half-second before and after are at least 35° [Hashemi, 2015].

We are assuming that the participant's head orientation is directly correlated to if he/she is watching the stimuli or not. This assumption is supported by the 'center bias' property in the gaze estimation literature [Li, 2013; Mannan, 1995]. In the future we plan to incorporate gaze estimation, not only to verify the child is looking at the screen but also to find where on the screen he/she is looking.

For each frame that the participant is deemed as looking at the screen we extract facial emotion information via a facial expression classifier [Hashemi, 2015; IntraFace]. We here group the results in 3 classes of emotions: Positive (Happy), Negative (Anger, Disgust, and Sad), and Neutral. These groups where selected because they represent emotions relevant to ASD, they are triggered by the carefully designed visual stimuli (which are designed to elicit positive emotions), and further guarantee robust coding by the automatic algorithm based on our previous studies [Hashemi, 2015]. The emotion profile is then computed aggregating and normalizing the emotion information over all the frames in the video, where the sum of the weights of the 3 emotions equals one. Lastly, we also compute a yaw angle profile, where the yaw angles are reduced to a histogram with 5 bins.

Autism risk. We examined autism risk (AS Risk) in the sample in three ways: 1. Caregiver report that child has an autism diagnosis in the Parental Concern Survey; 2. Child scored in the medium risk (total score 3-7) or high risk (total score 8-20) category on the M-CHAT-R/F; and 3. A composite variable of parent-reported autism diagnosis *and/or* high score on the M-CHAT (this variable was used as the outcome variable in video analyses for whole cohort).

Video extracted variables. As detailed above, we derived three emotion variables: percentage of positive emotion, negative emotion, and neutral expression. We created a categorical attention variable for children who did and did not attend in 90% or more of the frames.

Data and statistical analysis. Variables were extracted and exported to an Excel data set. Data presented here were analyzed using SAS 9.4, and the corresponding source code is available. Models of group differences were generated using generalized linear mixed models with backward selection to drop non-significant interaction terms. We used linear regression models adjusted for child age and sex to model the mean (standard error) of percent emotion or attention by autism risk status as measured by caregiver report and/or M-CHAT score. We also modeled the mean (standard error) of percent emotion or attention stratified by sex and adjusted for age. The significance value was evaluated at 0.05. To account for the impact of multiple comparisons we applied the Benjamini-Hochberg false discovery rate [Benjamini, 1995] set at 10% and have indicated in tables 4 and 5 which associations remain significant with this correction.

Study limitations

While a promising demonstration of the feasibility of video-based behavioral coding at home, this study has a number of limitations.

Our data are not representative of the general population in the US or of children at high risk for ASD. Our sample is skewed with higher participation of children with autism and boys (a

reflection that the rate of autism is four times higher in boys than girls [Christensen, 2016]). In addition, our assessment of autism risk in this study was based only on parent-report: parent-report of an autism diagnosis and/or the score on the parent-report M-CHAT. The incorporation of the study in environments such as clinics, schools, and community sites where autism status can be directly assessed can significantly alleviate these caveats.

To develop valid automatic behavior coding screening tools, we must conduct studies that compare the automatic coding in children who have comprehensive assessments using gold-standard tools such as the Autism Diagnostic Interview [ADI; Lord, 1994] and Autism Diagnostic Observational Scale [ADOS; Lord, 1989]. To establish the reliability and validity of our automatic coding for clinical and population-level screening, ResearchKit based studies will need to be coupled with such clinical studies. In parallel with Autism & Beyond, we conducted a study in pediatric primary care in which we administered the M-CHAT and our movie stimuli (in this case using an iPad) and then conducted full clinical ADOS assessments of the children who screened high on the M-CHAT [Campbell, 2016; Campbell, 2017; Campbell, 2017b]. These studies support the validity of our measurements, providing the starting point for the research here described. Key issues to address are how representative the samples of children are and the need to conduct full clinical assessments of both high and low risk children. Future work will also need to examine the specificity of automatically coded behaviors for autism risk through inclusion of children with other developmental disorders and mental health disorders that are associated with differences in emotion regulation, social behaviors, and attention. In other words, children with a large diversity of developmental and mental health disorders need to be included in the study to further understand both the feasibility of the approach and its sensitivity/specificity, including handling co-morbidity. All these extensive validations are part of our current efforts.

While caregivers are instructed not to actively trigger emotions and behaviors in the participants, and our clinical early work indicates this request is followed [Campbell, 2017b], it will be important to use the recorded video and sound to verify that the movie stimuli are the ones eliciting the child's response. Our face analysis could be applied to caregivers as well (we can easily detect and analyze multiple faces), not only to control for this but also to add the synchronization of child and caregiver emotions (if any) as a potential new marker.

This feasibility study concentrated on particular visual behaviors elicited by the designed movies. The literature is very clear that additional behaviors, from language to repetitive motor, are very important ASD markers as well, e.g., [Albinali, 2009; Amiri, 2017; Cheol-Hong, 2017; Gilchrist, 2017; Goodwin, 2011; Min, 2010; Nazneen, 2015; Oller, 2010; Xu, 2009; Xu, 2012]. These works show that a number of audio patterns such as child vocalization and syllabification, which can be automatically computed, can be critical ASD biomarkers. Similarly, stereotypical motor behaviors such as hand flapping and body rocking are important additional features to consider. Mobile devices and automatic coding can be used to address these as well, and even elicit them, and future generations of the developed app should consider including a larger spectrum of behaviors. Each different coded behavior will add to the needed screening scores.

The M-CHAT is a widely used autism screening measure. Psychometric data suggests that four out of ten children who screen high on the M-CHAT do not meet criteria for ASD, although most have another developmental disorder [Robins, 2013]. Our finding that 75% of M-CHAT high screeners in our study did not have a parent-reported autism diagnosis may reflect a low rate of early identification and/or M-CHAT false positives. Future studies on autism should include parent-report measures across the age range and, perhaps, multiple measures for comparison.

Another limitation is that participants must have access to an iPhone to participate in ResearchKit studies. In 2017, iOS represents 14.7% of the world wide smartphone operating share (https://www.idc.com/promo/smartphone-market-share/os) and 34% of the US

smartphone operating share (https://www.kantarworldpanel.com/global/smartphone-os-market-share/). We can enroll multiple subjects using one iPhone so that we can enroll families who might now have iOS devices in community settings. This approach addresses baseline enrollment but still does not address the needs for longitudinal research and on-going engagement with families. Deployment in hospitals, schools, and frequently visited facilities may be one way to address this challenge.

Study ethical issues

We are committed to defining the ethics of digital health research and population-level big data, particularly with vulnerable populations, which include children and people with developmental disability and mental illness.

Obtaining consent through an app without an in-person discussion of the risks and benefits limits our capacity to evaluate our participants' understanding of the potential risk associated with the study (see [Broome, 2003; Broome, 2008] for some research in these topics). Self-assessment of eligibility requirements also risks the inclusion of ineligible participants. The challenges posed with ResearchKit-type studies are central to all big data community-based research. The richness of these data offer new opportunities for scientific discovery, benefits which must be considered in light of risks to children's and caregivers' rights.

Our collection of video data of children in this study also raises specific ethical risks and challenges about children's rights to privacy since it is not possible to de-identify video data of children's faces. We consulted with ethicists at Duke Health and Duke University as we designed and conducted Autism & Beyond. To reduce risks, first we gave caregivers the option to upload only the facial landmarks from the recording of the child to mitigate the potential risks of uploading the full video. Second, in our consent, we stated that we would not share their data with researchers outside of our research group (the data resides in the Duke Health system).

Third, we told caregivers that they could contact us at any point and request that we delete all of their child's videos and data. The standard in most studies is that data already collected prior to withdrawal from a study can be retained; we went a step beyond this. Lastly, for full transparency, when the parent obtained a video of the child, a thumbnail copy of the video (side by side with the movie stimulus clip) was saved in the dashboard tab for the parent to review at any time.

There are also ethical issues related to identifying potential delays in a child's development or their risk for ASD without being able to provide clinical follow-up. In this study, we referred caregivers whose children scored greater than two on the M-CHAT to local early intervention resources and provided links to additional resources. Because the M-CHAT tool and scores are already available to caregivers on-line (https://www.autismspeaks.org/what-autism/diagnosis/mchat), we concluded that we were not adding additional risk by including it on the app.

Eligibility criteria

- Are you at least 18 years old?
    - Yes
    - No
- Do you have a child who is at least 1 year old and under 6 years old?
    - Yes
    - No
- Are you the parent or legal guardian of this child?
    - Yes
    - No
- Do you read and understand English
    - Yes
    - No
- Where do you live?
    - [Country Selector]

Family Background Survey

Introduction Page
- In this survey we will ask you about you and your child's family background.

Information Page
- Please tell us about your child.

Questions
- Is your child a boy or a girl?
    - Boy
    - Girl
- When was your child born?
    - [Text Entry]
- What is your child's ethnicity? Select all that apply.
    - Black / African American
    - Caucasian
    - Native American
    - Hawaiian or Other Pacific Islander
    - Middle Eastern
    - Caribbean
    - South Asian
    - East Asian
    - Hispanic, Latino or of Spanish origin
    - Other

Information Page
- Please tell us about yourself.

Questions
- What is your relationship to your child?
    - Parent
    - Legal Caregiver
- What is your gender?
    - Male
    - Female
    - Other (only 1-is this right?)
    - Prefer not to answer (is this right?)
- When were you born?
    - [Text Entry]
- What is your ethnicity? Select all that apply.
    - Black / African American
    - Caucasian
    - Native American
    - Hawaiian or Other Pacific Islander
    - Middle Eastern
    - Caribbean
    - South Asian
    - East Asian
    - Hispanic, Latino or of Spanish origin
    - Other
- What is the highest level of school that you have completed or highest degree you have received?

  - Doctoral degree (e.g., PhD, MD, JD, etc)
  - Master's degree
  - Bachelor degree
  - Associate degree
  - Some college but no degree
  - High School Diploma / GED
  - Some high school
- Which of the following categories best describes your employment status?
  - Employed working 40 or more hours per week
  - Employed working 1 - 39 hours per week
  - Not employed, looking for work outside of the home
  - Self-employed
  - Stay-at-home caregiver not working outside of the home
  - Retired
  - Disabled, unable to work outside of home
  - Other
- What is your current relationship status?
  - Single, never married
  - Married or domestic partnership
  - Widowed
  - Divorced
  - Separated
  - Other
- How did you learn about the Autism & Beyond Study (select all that apply)?
  - Social media
  - Email from a friend(only 1 is this right?)
  - Health professional or health center
  - Advertisement (e.g., in iTunes, print media)
  - Searching on-line (e.g., Google, etc)
  - General media coverage (e.g., news study, radio, print, TV, on-line)
- May we contact you by e-mail about possible participation in future studies?
  - Yes
  - No

Information Page

- Please tell us about your family.

Questions

- How many children live in your household?
  - [Text Entry]
- What is the primary language spoken in your home?
  - [Text Entry]

Information Page

- Thank you for completing this survey. Please click Done to save your responses.

Parental Concerns Survey

Introduction Page

- In this survey we will ask you about any concerns you have about your child's development. We will also ask about any services your child has received.

Questions

- Do you have any concerns about your child's use of words or gestures to communicate with others?
  - Yes
  - No
  - Unsure
- Do you have any concerns about your child's hearing?
  - Yes
  - No
  - Unsure
- Do you have any concerns about your child's motor development?
  - Yes
  - No
  - Unsure
- Do you have any concerns about your child's social interactions?
  - Yes
  - No
  - Unsure
- Do you have concerns about your child's social interactions with you?
  - Yes
  - No
  - Unsure
- Do you have concerns about your child's social interactions with other adults?
  - Yes
  - No
  - Unsure
- Do you have concerns about your child's social interactions with other children?
  - Yes
  - No
  - Unsure
- Do you have any concerns about your child's sleep?
  - Yes
  - No
  - Unsure
- Do you have any concerns about the way your child manages emotions?
  - Yes
  - No
  - Unsure
- Do you have any concerns about the way your child manages anxiety / fear?
  - Yes
  - No
  - Unsure
- Do you have any concerns about the way your child manages sadness?
  - Yes
  - No

  - Unsure
- Do you have any concerns about the way your child manages anger?
  - Yes
  - No
  - Unsure
- Do you have any concerns about your child's temper tantrums?
  - Yes
  - No
  - Unsure
- Do you have any concerns about your child paying attention?
  - Yes
  - No
  - Unsure
- Do you have any concerns about your child's activity level?
  - Yes
  - No
  - Unsure
- Have you talked with others about your concerns?
  - Yes
  - No
  - Unsure
- Have you talked with others about your concerns?
  - With your partner
  - With other family member(s)
  - With your child's medical doctor?
  - With a developmental or medical health professional
  - With a teacher or daycare provider
  - With a friend
  - With clergy
- Has your child ever had a developmental evaluation or a mental health evaluation?
  - Yes
  - No
  - Unsure
- When was that?
  - Month, day,year

- Has your child been diagnosed with any of the following?
  - Language Delay
  - Developmental Delay
  - Autism Spectrum Disorder
  - Attention deficit / Hyperactivity Disorder
  - Behavior Disorder
  - Depression
  - Genetic Disorder
  - Chronic Mental Illness
  - Other (the only other -is this right)
  - No
- Have you faced barriers trying to get help for your child?
  - Yes
  - No

  - NOT FOUND
- Which of the following barriers have you faced?
  - Can't get an appointment
  - On a waiting list
  - Costs too much
  - Don't have transportation
  - Not available where I live
  - Language barrier
  - Insurance does not cover
  - Feel embarrassed
  - Feel scared
  - Feel confused
  - Other
- Do you feel that you have the energy to handle challenges that your child faces?
  - Never
  - Not often
  - Sometimes
  - Most of the time
  - Always
- How happy are you today?
  - Completely Unhappy
  - [Sliding Scale Entry]
  - Completely Happy
- Is there anything else that you would like to share with us about your child or family?
  - [Text Entry]
- Is there anything else that you would like to share with us about being a parent?
  - [Text Entry]

Information Page

- Thank you for completing this survey. Please click Done to save your responses.

Temper Tantrum Survey

Introduction Page

- Duke Temper Tantrum Survey
- In this survey, we will ask you about your child's behavior.

Questions

- In the last month, has your child had a temper tantrum?
  - Yes
  - No
- In the last month, has your child had a temper tantrum nearly every day?
  - Yes
  - No
- In the last month during a temper tantrum has your child hit someone?
  - Yes
  - No
- In the last month during a temper tantrum has your child bitten someone?
  - Yes
  - No
- In the last month during a temper tantrum has your child kicked someone?
  - Yes
  - No
- In the last month during a temper tantrum has your child hit him or herself?
  - Yes
  - No
- In the last month during a temper tantrum has your child bitten him or herself?
  - Yes
  - No
- In the last month during a temper tantrum has your child broken an object (like a toy or a cup)?
  - Yes
  - No

Information Page

- Thank you for completing this survey. Please click Done to save your responses.

Bibliography

[Albinali, 2009] F. Albinali, M. S. Goodwin, and S. S. Intille, Recognizing stereotypical motor movements in the laboratory and classroom: A case study with children on the autism spectrum, *Proceedings of the International Conference on Ubiquitous Computing*, pp. 71-80, Florida, 2009.

[Amiri, 2017] A. M. Amiri, et al., WearSense: Detecting autism stereotypic behaviors through smartwatches, *Healthcare*, Basel, 2017.

[Benjamini, 1995] Y. Benjamini and Y. Hochberg, Controlling the false discovery rate: A practical and powerful approach to multiple testing, *Journal of the Royal Statistical Society, Series B (Methodological)* 57:1, pp. 289–300, 1995.

[Bot, 2016] B. Bot, C Suver, E. Chaibub Neto, M. Kellen, A. Klein, C. Bare, M. Doerr, A. Pretap, J. Wilbanks, E. R. Dorsey, S. H. Friend, and A. D. Triester, The mPower study, Parkinson disease mobile data collected using ResearchKit, *Scientific Data* 3, 2016.

[Brooks, 2016] B. A. Brooks, K. Haynes, J. Smith, T. McFadden, and D. L. Robins, Implementation of web-based Autism screening in an urban clinic, *Clin. Pediatr.* **55**, pp. 927-934, 2016.

[Broome, 2003] M.E. Broome, E. Kodish, G. Geller, and L. Siminoff, Children in research: New perspectives and practices for informed consent. *IRB: Ethics & Human Research* **25(5)**, S20-S23, 2003.

[Broome, 2008] M.E. Broome, Intervention research in bioethics. In L. Jacoby and L. Siminoff (Eds.), Empirical methods for bioethics: A primer. *Advances in Bioethics* **11** (203-217). Oxford, UK: JAI Press

[Campbell, 2016] K. Campbell, K., Hashemi, J., Espinosa, S., Marsan, S., Schaich Borg, J., Harris, A., Chang, Z., Qiu, Q., Tepper, M., Calderbank, R., Baker., J.P., Sapiro, G., Egger, H., and Dawson, G. Computer vision detects delayed social orienting in toddlers with autism. *2016 Annual Meeting of the International Society for Autism Research*, May 2016, Baltimore, MD.

[Campbell, 2017] K. Campbell, K.L.H. Carpenter, S. Espinosa, J. Hashemi, Q. Qiu, M. Tepper, R. Calderbank, G. Sapiro, H.L. Egger, J.P. Baker, and G. Dawson, Use of a digital M-CHAT-R/F to improve quality of screening for autism, *Journal of Pediatrics*, 183: 133-139, 2017.

[Campbell, 2017b] K. Campbell, K.L.H. Carpenter, S. Espinosa, J. Hashemi, Q. Qiu, M. Tepper, R. Calderbank, G. Sapiro, H.L. Egger, J.P. Baker, and G. Dawson. Computer vision analysis detects inconsistent and delayed social orienting in toddlers with autism, *Autism: International Journal of Research and Practice*, 2018, to appear.

[Cheol-Hong , 2017] M. Cheol-Hong, Automatic detection and labeling of self-stimulatory behavioral patterns in children with Autism Spectrum Disorder*, Conf Proc IEEE Eng Med Biol Soc, 2017*, pp. 279-282, 2017.

[Chlebowski, 2013] C. Chlebouwski, D. L. Robins, M. L. Barton, and D. Fein, Large-scale use of the modified checklist for Autism in low-risk toddlers, *Pediatrics* 131, pp. 1121-1127, 2013.

[Christensen, 2016] D. L. Christensen *et al.* Prevalence and characteristics of autism spectrum disorder among children aged 8 years -- Autism and Developmental Disabilities Monitoring Network, 11 Sites, United States, 2012. *Morbidity and Mortality Weekly Report. Surveillance Summaries (Washington, D.C. : 2002)* **65**, pp. 1-23, 2016.

[Dawson, 2004] G. Dawson, K. Toth, R. Abbott, J. Osterling, J. Munson, A. Estes, and J. Liaw, Early social attention impairments in autism: social orienting, joint attention, and attention to distress, *Dev Psychol*. **40(2)**, pp. 271-83, 2004.

[Egger, 2003] H. Egger, *Temper tantrums and preschool mental health*. Symposium. Presented at the 50th American Academy of Child and Adolescent Psychiatry Annual Meeting, Miami, Florida, 2003.

[Gilchrist, 2017] K. H. Gilchrist et al., Automated detection of repetitive motor behaviors as an outcome measurement in intellectual and developmental disabilities, *Journal of Autism and Developmental Disorders*, 2017.

[Goodwin, 2011] M. S. Goodwin et al., Automated detection of stereotypical motor movements, *Journal of Autism and Developmental Disorders* **41(**6), pp. 770-782, 2011.

[Hashemi, 2015] J. Hashemi, K. Campbell, K. L.H. Carpenter, A. Harris, Q. Qiu, M. Tepper, S. Espinosa, J. Schaich Borg, S. Marsan, R. Calderbank, J. P. Baker, H. L. Egger, G. Dawson, and G. Sapiro, A scalable app for measuring autism risk behaviors in young children: A technical validity and feasibility study, *MobiHealth* 2015, London, October 2015.

[Hashemi, 2015b] J. Hashemi, Q. Qiu, and G. Sapiro, Cross-modality pose-invariant facial expression, *IEEE International Conference Image Processing*, Quebec City, Canada, September 2015.

[IntraFace] http://www.humansensing.cs.cmu.edu/intraface/

[Jones, 2016] E.J.H. Jones, K. Venema, R. Earl, R. Lowy, K. Barnes, A. Estes, G. Dawson, and SJ Webb, Reduced engagement with social stimuli in 6-month-old infants with later autism spectrum disorder: A longitudinal prospective study of infants at high familial risk, *Journal of Neurodevelopmental Disorders* **18:8**, 2016

[Jones, 2017] EJ Jones, G. Dawson, J. Kelly, A. Estes, SJ Webb, Parent-delivered early intervention in infants at risk for ASD: Effects on electrophysiological and habituation measures of social attention, *Autism Research*, **10(5)**, pp. 961-972, 2017.

[Kirkovski, 2013] M. Kirkovki, P. G. Enticott, and P. B. Fitzgerald, A review of the role of female gender in autism spectrum disorders, *J. Autism Developmental Disorders* 43, pp. 2584-2603, 2013.

[Kumm, 2017] A. J. Kumm, K. Campbell, S. Marsan, J. Hashemi, S. Espinosa, R. Bloomfield, G. Dawson, G. Sapiro, H. Egger, and P. J. de Vries, Feasibility of a smartphone application to identify young children at risk for autism spectrum disorder in a low-income, community setting in South Africa, *IMFAR,* San Francisco, May 2017.

[Li, 2013] Y. Li, A. Fathi, and J. Rehg. Learning to predict gaze in egocentric video, In *ICCV*, Sydney, Australia, 2013.

[Lord, 1989] C. Lord *et al.*, Autism diagnostic observation schedule: a standardized observation of communicative and social behavior, *J Autism Dev Disord, **19***(**2**), pp. 185-212, 2989.

[Lord, 1994] C. Lord, M. Rutter, and A. Le Couteur, Autism Diagnostic Interview-Revised: a revised version of a diagnostic interview for caregivers of individuals with possible pervasive developmental disorders, *J Autism Dev Disord* **24(5),** pp. 659-685, 1994.

[Mannan, 1995] S. Mannan, K. Ruddock, and D. Wooding, Automatic control of saccadic eye movements made in visual inspection of briefly presented 2-d images, *Spatial Vision*, vol. 9, no. 3, pp. 363–386, 1995.

[Min, 2010] C. H. Min and A.H. Tewfik, Automatic characterization and detection of behavioral patterns using linear predictive coding of accelerometer sensor data, *Conf Proc IEEE Eng Med Biol Soc 2010*, pp. 220-223, 2010

[Nazneen, 2015] N. Nazneen, A. Rozga, C. J. Smith, R. Oberleitner, G. D. Abowd, and R. I. Arriaga, A novel system for supporting autism diagnosis using home videos: Iterative development and evaluation of system design, *JMIR Mhealth Uhealth* 3(2), 2015.

[Oller, 2010] D. K. Oller et al., Automated vocal analysis of naturalistic recordings from children with autism, language delay, and typical development*, Proc Natl Acad Sci U S A,* 107(30), pp. 13354-13359, 2010.

[Robins, 2013] D. L. Robins, K. Casagrande, M. Barton, C.-M. A. Chen, T. Dumont-Mathieu, and D. Fein, Validation of the Modified Checklist for Autism in Toddlers, Revised With Follow-up (M-CHAT-R/F), *Pediatrics* 133, pp. 37-45, 2013.

[Webster, 2017] D. E. Webster, C. Suver, M. Doerr, E. Munts, L. Domenico, T. Petrie, S. A. Leachman, A. D. Triester, and B. M. Bot, The Mole Mapper Study, mobile phone skin imaging and melanoma risk data collected using ResearchKit, *Scientific Reports,* 4, 2017.

[Xu, 2009] D. Xu et al., Child vocalization composition as discriminant information for automatic autism detection, *Proc IEEE Eng Med Biol Soc 2009,* pp. 2518-2522, 2009.

[Xu, 2012] D. Xu, J. Gilkerson, and J.A. Richards, Objective child behavior measurement with naturalistic daylong audio recording and its application to autism identification*, Proc IEEE Eng Med Biol Soc 2012,* pp. 3708-3711, 2012.

Figures, tables, and videos legends

Figure S1: Caregiver's app engagement. Flow diagram of caregivers' engagement with the Autism & Beyond study.

Figure S2: Enrollment distribution. Distribution of enrollment in the first months of the study. Participants learned about the study through multiple sources including social media (n=957, 54.7%), advertising in iTunes or other media (n=618, 35.3%), email from a friend (n=126, 7.2%), health professional or health center (n=45, 2.3%), and searching on line (n=123, 7.0%). Participants could choose multiple avenues; nearly three quarters learned about the study from one single source (n=1266, 72.4%) with the top single source being social media (n=869, 49.7% of sample).

Figure S3: E-consent screen-shots. Examples of the screen-shots representing e-Consent information provided to potential participants before they agree to enroll. The content and screens for the e-Consent for approved by the Duke University Medical Center IRB.

Figure S4: The four tabs in the app. Once enrolled in the study, participants had access to four tabs: activities, dashboard, learn, and profile.

Figure S5: Feedback examples. Examples of the feedback provided to caregivers.

Table S1. Mean percent emotion. This table shows the mean percent emotion (standard error) by video clip and autism spectrum risk status based on M-CHAT score, overall and among boys, adjusted for child age in months

Video S1: Movie stimuli. Segments of the movie stimul (see Video S1).

Video S2: Example of emotion analysis. Example of automatic emotion analysis and head position for a child watching

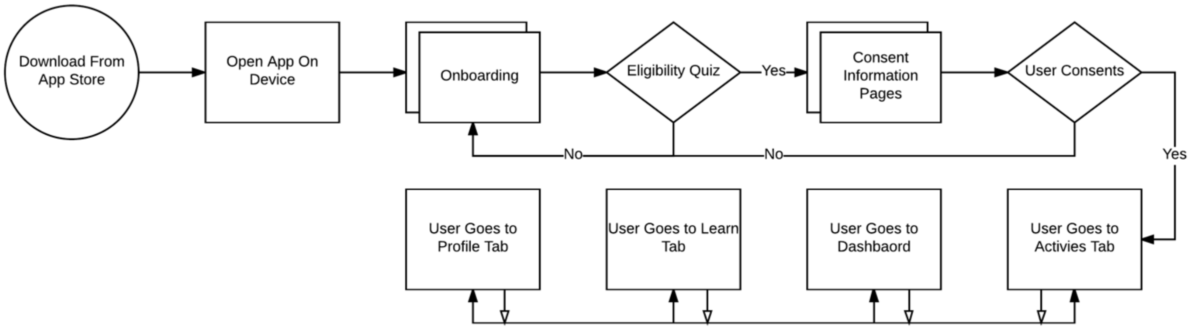
Download From App Store
Open App On Device
Onboarding
Eligibility Quiz
Yes
Consent Information Pages
User Consents
No
No
Yes
User Goes to Profile Tab
User Goes to Learn Tab
User Goes to Dashbaord
User Goes to Activies Tab

Figure S1

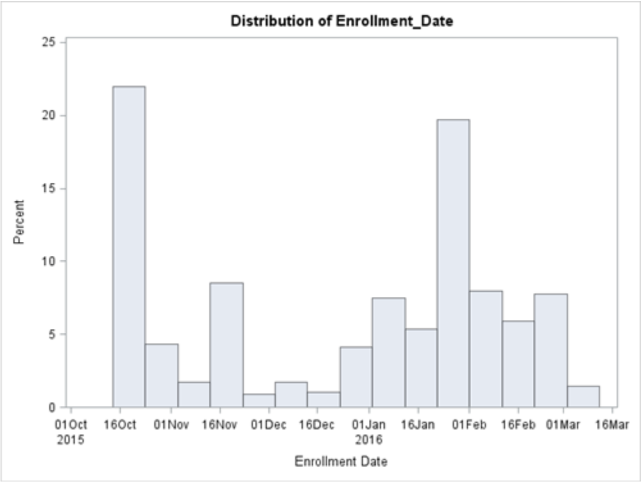
Distribution of Enrollment_Date
25
20
15
10
5
0
Percent
01Oct 2015
16Oct
01Nov
16Nov
01Dec
16Dec
01Jan 2016
16Jan
01Feb
16Feb
01Mar
16Mar
Enrollment Date

Figure S2

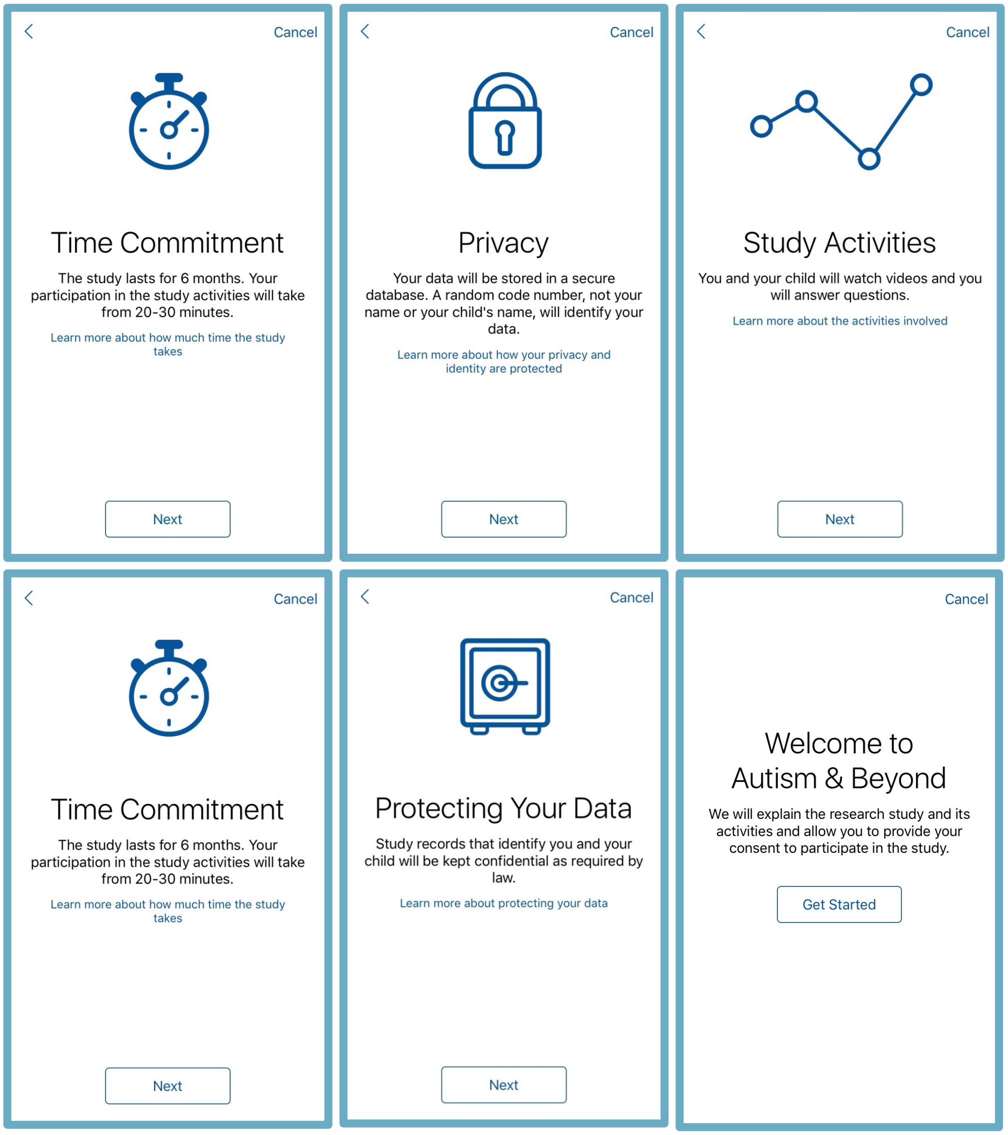
Cancel
Time Commitment
The study lasts for 6 months. Your participation in the study activities will take from 20-30 minutes.
Learn more about how much time the study takes
Next
Cancel
Privacy
Your data will be stored in a secure database. A random code number, not your name or your child's name, will identify your data.
Learn more about how your privacy and identity are protected
Next
Cancel
Study Activities
You and your child will watch videos and you will answer questions.
Learn more about the activities involved
Next
Cancel
Time Commitment
The study lasts for 6 months. Your participation in the study activities will take from 20-30 minutes.
Learn more about how much time the study takes
Next
Cancel
Protecting Your Data
Study records that identify you and your child will be kept confidential as required by law.
Learn more about protecting your data
Next
Cancel
Welcome to Autism & Beyond
We will explain the research study and its activities and allow you to provide your consent to participate in the study.
Get Started

Figure S3

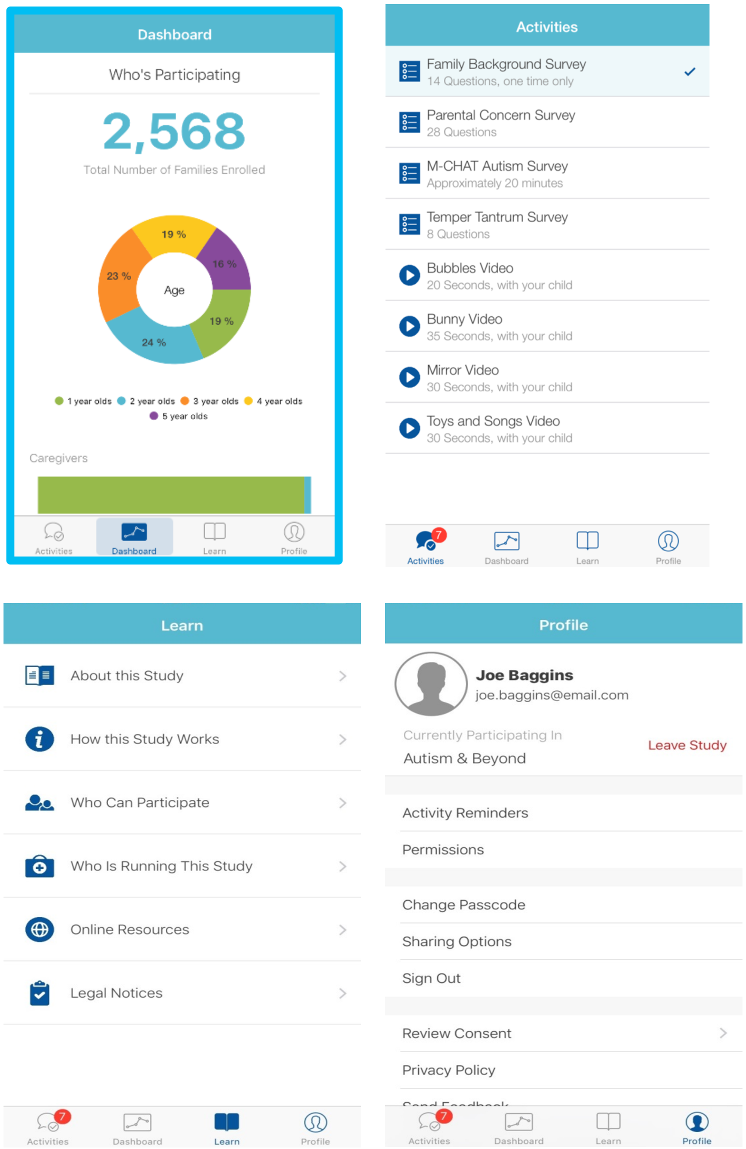
Dashboard
Who's Participating
2,568
Total Number of Families Enrolled
19 %
16 %
23 %
Age
19 %
24 %
1 year olds
2 year olds
3 year olds
4 year olds
5 year olds
Caregivers
Activities
Dashboard
Learn
Profile
Activities
Family Background Survey
14 Questions, one time only
Parental Concern Survey
28 Questions
M-CHAT Autism Survey
Approximately 20 minutes
Temper Tantrum Survey
8 Questions
Bubbles Video
20 Seconds, with your child
Bunny Video
35 Seconds, with your child
Mirror Video
30 Seconds, with your child
Toys and Songs Video
30 Seconds, with your child
Learn
About this Study
How this Study Works
Who Can Participate
Who Is Running This Study
Online Resources
Legal Notices
Profile
Joe Baggins
joe.baggins@email.com
Currently Participating In
Autism & Beyond
Leave Study
Activity Reminders
Permissions
Change Passcode
Sharing Options
Sign Out
Review Consent
Privacy Policy

***Figure* S4**

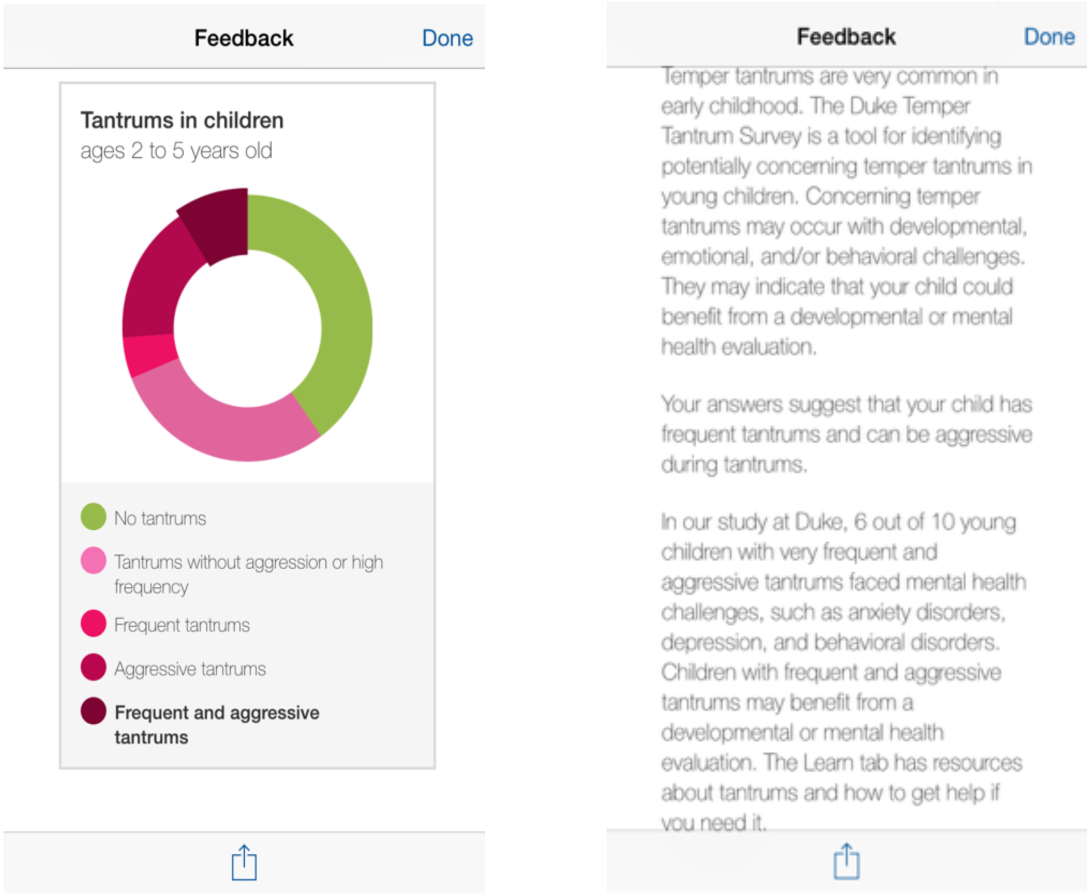
Feedback
Done
Tantrums in children
ages 2 to 5 years old
No tantrums
Tantrums without aggression or high frequency
Frequent tantrums
Aggressive tantrums
Frequent and aggressive tantrums
Feedback
Done
Temper tantrums are very common in early childhood. The Duke Temper Tantrum Survey is a tool for identifying potentially concerning temper tantrums in young children. Concerning temper tantrums may occur with developmental, emotional, and/or behavioral challenges. They may indicate that your child could benefit from a developmental or mental health evaluation.
Your answers suggest that your child has frequent tantrums and can be aggressive during tantrums.
In our study at Duke, 6 out of 10 young children with very frequent and aggressive tantrums faced mental health challenges, such as anxiety disorders, depression, and behavioral disorders. Children with frequent and aggressive tantrums may benefit from a developmental or mental health evaluation. The Learn tab has resources about tantrums and how to get help if you need it.

Figure S5

Table S1. Mean percent emotion (standard error) by video clip and autism spectrum risk status based on M-CHAT score, overall and among boys, adjusted for child age in months

		All Children							Boys							
		High (8-20)		Med (3-7)		Low (0-2)				High (8-20)		Med (3-7)		Low (0-2)		
Movie Clip	N	Mean	SE	Mean	SE	Mean	SE	P-value	N	Mean	SE	Mean	SE	Mean	SE	P-value
Neutral Emotion																
Bubbles	180	37.1	4.6	35.3	4.2	27.8	2.7	0.11	130	42.0	5.0	37.3	4.7	28.1	3.1	0.04
Bunny	146	38.0	4.8	31.5	4.4	27.0	2.7	0.11	108	42.5	4.9	36.3	4.6	27.9	3.1	0.03
Mirror	126	38.5	4.8	30.9	4.5	28.4	2.6	0.16	94	38.8	5.4	34.8	4.5	28.0	3.0	0.16
Toys & Songs	124	35.1	5.6	36.6	5.4	35.1	3.3	0.97	95	34.3	5.8	36.3	5.3	32.8	3.5	0.86
Positive Emotion																
Bubbles	180	15.8	4.7	29.1	4.3	27.9	2.8	0.05	130	14.1	5.1	29.4	4.7	29.1	3.1	0.03
Bunny	146	15.8	4.9	28.3	4.5	24.0	2.8	0.14	108	11.1	4.9	26.7	4.6	27.9	3.0	0.01
Mirror	126	23.2	4.7	25.6	4.5	30.8	2.6	0.27	94	21.0	5.3	24.8	4.4	32.7	3.0	0.10
Toys & Songs	124	17.8	5.2	28.8	5.0	21.9	3.1	0.25	95	13.2	5.3	24.7	4.8	23.7	3.2	0.20
Negative Emotion																
Bubbles	180	47.2	4.6	35.6	4.2	44.3	2.7	0.11	130	44.0	5.1	33.3	4.8	42.8	3.2	0.20
Bunny	146	46.2	5.2	40.3	4.7	49.0	3.0	0.26	108	46.3	5.3	37.0	5.0	44.2	3.3	0.37
Mirror	126	38.3	5.0	43.5	4.8	40.8	2.8	0.73	94	40.2	5.5	40.4	4.7	39.3	3.1	0.98
Toys & Songs	124	47.2	5.9	34.6	5.8	42.9	3.5	0.25	95	52.5	6.4	39.1	5.8	43.5	3.8	0.29
Attention																
Bubbles	185	91.4	3.9	91.1	3.6	88.8	2.3	0.77	131	95.2	3.9	91.0	3.7	89.2	2.4	0.43
Bunny	147	94.5	4.2	89.5	3.8	88.2	2.4	0.40	109	93.8	4.1	93.4	3.9	87.5	2.5	0.28
Mirror	126	88.0	4.0	86.7	3.8	88.0	2.2	0.95	94	87.1	4.1	88.2	3.5	87.6	2.3	0.98
Toys & Songs	127	89.6	5.0	93.0	4.8	90.9	2.9	0.87	97	88.5	5.6	90.2	5.1	89.0	3.3	0.97

Key: P values did not remain significant after applying the Benjamini-Hochberg procedure [Benjamini, 1995] with a 10% false discovery rate to account for the impact of multiple comparisons;
